# Serial assessment of inflammatory biomarkers as a prognostic factor for first-line treatment outcomes in patients with metastatic colorectal cancer: single-center retrospective study

**DOI:** 10.3389/fonc.2026.1737930

**Published:** 2026-01-27

**Authors:** Nikša Librenjak, Gordan Adžić, Domina Kekez, Irma Goršić, Juraj Prejac, Stjepko Pleština

**Affiliations:** 1 Department of Oncology, University Hospital Centre Zagreb, Zagreb, Grad Zagreb, Croatia; 2 School of Dental Medicine, University of Zagreb, Zagreb, Grad Zagreb, Croatia; 3 School of Medicine, University of Zagreb, Zagreb, Grad Zagreb, Croatia

**Keywords:** first-line chemotherapy, metastatic colorectal cancer, neutrophil-to-lymphocyte ratio (NLR), systemic immune-inflammation index (SII), systemic inflammation, treatment outcomes

## Abstract

**Background:**

Metastatic colorectal cancer (mCRC) is a major cause of cancer-related mortality, with limited curative options despite advances in targeted therapies. Reliable prognostic biomarkers are needed to guide treatment. Inflammatory markers such as the neutrophil-to-lymphocyte ratio (NLR) and systemic immune-inflammation index (SII) may predict poor outcomes. This study evaluated the prognostic value of serial NLR and SII assessments in first-line mCRC therapy.

**Methods:**

In this single-center, retrospective, observational study, we obtained data on 258 patients who started first-line systemic chemotherapy from the beginning of 2016 until the end of 2018. Baseline (pretreatment) and 3-month post-treatment values of NLR and SII were used to determine their prognostic value for treatment outcomes, which were measured as progression-free survival (PFS) and overall survival (OS).

**Results:**

Patients were divided by pretreatment median NLR (3.19) and SII (810). Low NLR was associated with longer PFS (15.4 vs. 9.5 months, p=0.031) and OS (39.1 vs. 27.2 months, p=0.002). After 3 months, PFS difference increased (14.3 vs. 4.5 months, p<0.001); OS was longer but not significantly (35.3 vs. 20.1 months, p=0.14). Low SII after three months of therapy was linked to improved PFS (14.1 vs. 6.8 months, p<0.001) and OS (36.4 vs. 21.6 months, p=0.001), while at baseline assessment it was associated with longer OS (39.4 vs. 27.1 months, p=0.003), but not with PFS (13.9 vs. 10.6 months, p=0.11). Multivariate analysis showed pretreatment NLR and SII were independent prognostic factors for OS, but not PFS. Post-treatment NLR was prognostic for PFS only, while post-treatment SII was prognostic for both OS and PFS.

**Conclusions:**

Serial evaluation of NLR and SII could identify patients who are at increased risk of poor survival outcomes.

## 1 Introduction

Colorectal cancer (CRC) is the second leading cause of cancer-related mortality in men and the third in women within the European Union (1). At the time of diagnosis, 15 to 25% of patients already have metastatic disease, and an additional 20% develop metastases during follow-up. Despite advancements in treatment over the past decade, metastatic CRC (mCRC) remains incurable, with a median overall survival (OS) exceeding 30 months (2). mCRC is a heterogeneous disease, and the European Society for Medical Oncology (ESMO) guidelines emphasize the importance of tailoring therapy based on patient and tumor characteristics (3, 4). First and second-line treatments are based on chemotherapy regimens incorporating three cytotoxic agents: 5-fluorouracil, irinotecan, and oxaliplatin. These are used either as monotherapy or in combination with targeted therapy added depending on RAS/BRAF status (5–8). Treatment selection depends on the patient’s general condition, treatment goals, surgical resectability, and microsatellite instability (MSI) and RAS/BRAF molecular testing results (9). Inflammation plays a crucial role in the development of various diseases, including cancer, highlighting the need to explore markers that can assess systemic inflammation. Chronic inflammation, characterized by immune activity and the presence of cytokines, growth factors, and chemokines, can promote tumor initiation and support tumor growth, survival, and angiogenesis (10). Recently, there has been increased recognition of the tumor microenvironment’s significance, with the inflammatory response being considered a key factor in tumor progression and metastasis (11). Furthermore, systemic inflammation has been identified as a negative prognostic indicator in many malignancies (12). The systemic inflammatory response can be evaluated using blood cell counts and ratios, including the neutrophil-to-lymphocyte ratio (NLR) and the systemic immune-inflammation index (SII), as well as markers such as C-reactive protein (CRP) and serum albumin. NLR and SII are simple, low-cost biomarkers derived from routine complete blood counts (CBCs) and have been found to have prognostic value, correlating with poorer survival outcomes in patients with various tumor types, including CRC (13, 14). Elevated NLR is not specific to cancer and can be seen in various conditions, including infections, chronic inflammatory diseases, and cardiovascular events (15). However, there is currently no consensus on specific threshold values for NLR, and its prognostic significance varies across different stages and histological types of solid tumors (13, 15), as well as concerning clinical data. In stage II–III CRC a high NLR has been associated with poorer outcomes, likely reflecting greater tumor proliferative activity. In contrast, its predictive value in stage IV disease remains uncertain (16). On the other hand, the prognostic role of the SII in malignant disease has received less attention in research, especially in stage IV (14). In CRC patients who underwent surgery, SII was identified as an independent predictive factor for OS, demonstrating superior diagnostic performance compared to NLR (17, 18). Limited research has focused on the clinical application of NLR and SII specifically in mCRC, particularly in the context of first-line treatments. Consequently, there is a lack of data regarding the use of NLR and SII for patient selection and monitoring in modern treatment protocols, which include targeted therapies (19–21).

## 2 Methods

### 2.1 Patients and data collection

This study is a single-center, observational, retrospective analysis of patients diagnosed with mCRC who initiated chemotherapy at the Department of Oncology, University Hospital Centre Zagreb (UHC Zagreb), from January 1, 2016, to December 31, 2018. Included patients were older than 18 years with histologically confirmed mCRC and had not received any prior systemic treatment for metastatic disease. Patients with concomitant chronic inflammatory disease, those receiving glucocorticoid therapy, and those with a history of chemotherapy for a previous malignancy or active concurrent malignancy were not included in the study. Ethical approval for this research was obtained from the Ethics Committee of the UHC Zagreb. Data was extracted from medical records and includes encompassing factors such as age, sex, primary tumor location (with right-sided tumors defined up to the lienal flexure), disease stage at colorectal cancer diagnosis, primary tumor resection, adjuvant treatment, the extent of metastatic burden, KRAS, NRAS and BRAF mutational statuses, commencement date and duration of the initial treatment regimen, specifics of chemotherapy protocols and the application of targeted therapy, response to chemotherapy, use of local ablative therapy (surgery for metastases, microwave ablation - MWA or stereotactic body radiotherapy – SBRT), and CBC parameters before treatment initiation and after three months of treatment.

### 2.2 Study outcomes and definition

Reevaluation of the disease occurred at three-month intervals using a computed tomography (CT) scan, with criteria according to the Response Evaluation Criteria in Solid Tumors version 1.1 (RECIST 1.1) (22). OS was defined as the time from the commencement of the first cycle of chemotherapy for first-line metastatic disease to death from any cause. Progression-free survival (PFS) is defined as the time from the initiation of chemotherapy to documented disease progression or death from any cause. The Eastern Cooperative Oncology Group performance status (ECOG PS) was consistently assessed and monitored throughout the treatment course (23). Exclusion criteria for CBC parameter collection included treatment with granulocyte growth factors and/or antibiotics within 14 days prior to sampling. The NLR is calculated by dividing the absolute number of neutrophils by the absolute number of lymphocytes, while SII is calculated as the product of the absolute number of platelets and neutrophils divided by the absolute number of lymphocytes. These ratios were determined before the initiation of metastatic disease treatment and at each subsequent disease reevaluation.

### 2.3 Statistical analysis

Survival estimates (PFS and OS) were generated using the Kaplan-Meier method, presented as survival curves, and compared using the log-rank test. Normality of data distribution was assessed with the Shapiro–Wilk test and group comparisons were performed using the t-test or Mann–Whitney ***U*** test depending on the normality of distribution. Statistical significance was set at P = 0.05. Data on PFS were censored at a 36-month cut-off, and for OS at 60 months. Univariate and multivariate Cox multiple regression analyses were used to examine the association between survival and NLR and SII, and other influencing factors listed above. Results were expressed as hazard ratios (HR) with 95% confidence interval (CI), with statistical significance set at a confidence level of P<0.05. Data was analyzed using the JASP Team (2024). JASP (Version 0.95.1).

## 3 Results

### 3.1 Patient population baseline characteristics

**Figure 1:**
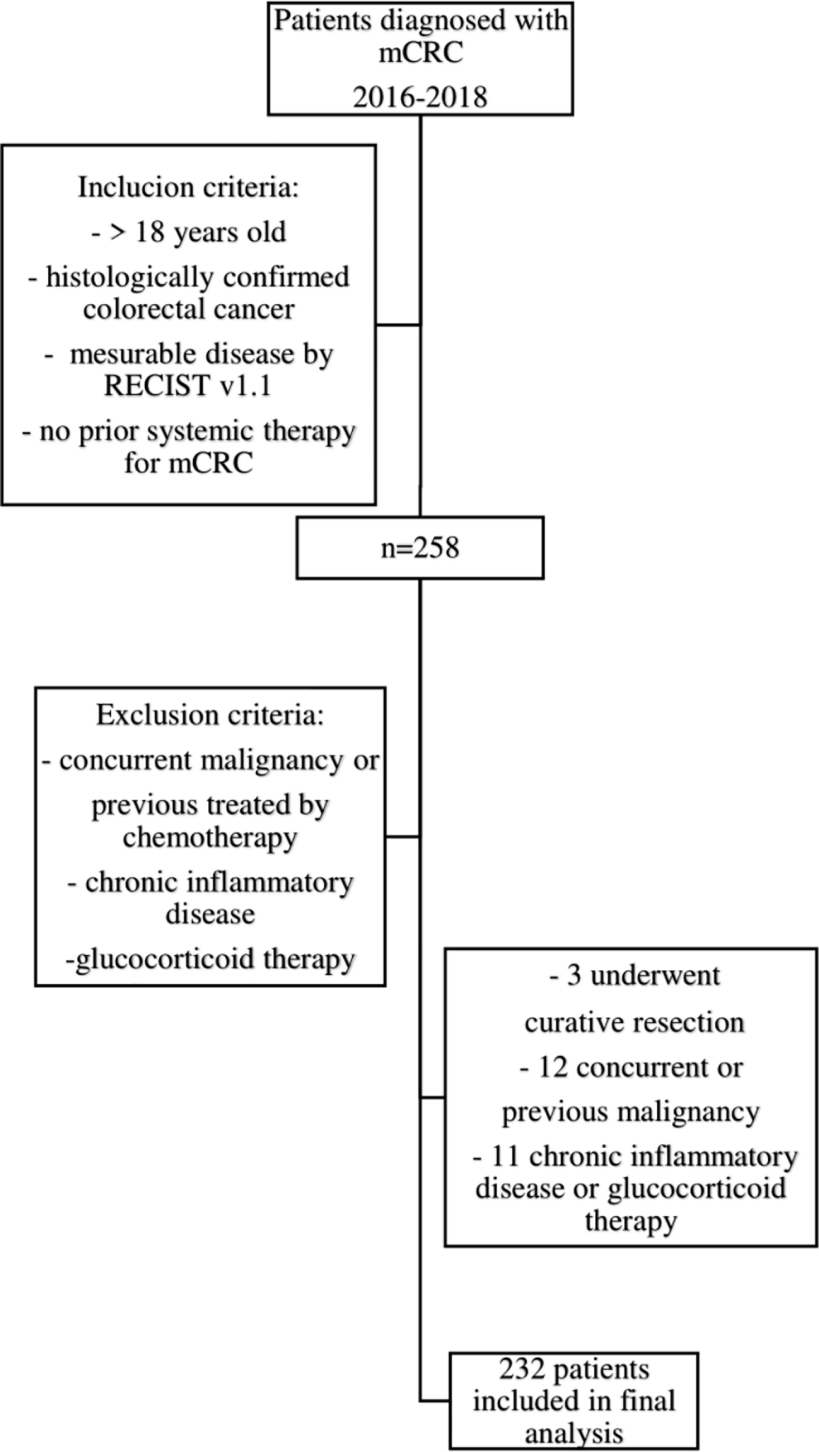
CONSORT diagram. Flowchart of patients’ disposition for the study.

This study aimed to examine the prognostic role of NLR and SII in the first-line treatment of mCRC. During the inclusion period, 258 patients started first-line treatment for mCRC, and 232 met the inclusion criteria (**Figure 1**). The median age was 63. The patient population exhibited a slight male predominance (61% male, 39% female). A majority of patients were under 65 years of age (58%), with 42% being 65 years or older. Tumor location was predominantly left-sided (81%), with right-sided tumors accounting for 19% of cases. Regarding RAS mutational status, 40.9% of patients were wild-type, while 42.7% had mutated RAS; 13.8% had not undergone testing, and 2.6% had BRAF mutations. The majority of patients presented with a good ECOG PS of 0 (80%), while 20% had an ECOG PS of 1 or higher. The remaining patient’s baseline characteristics are summarized in **Table 1**. Based on the pre-treatment median, NLR and SII were divided into groups, and differences between them were tested using the χ2 test. Groups were numerically well-balanced with no significant differences observed, with the exception of peritoneal and liver involvement based on pre-treatment NLR. Patients with low pre-treatment NLR and SII had statistically significantly higher disease control rate (DCR), defined as partial response (PR) or stable disease (SD) (**Table 2**). The median PFS and OS for the whole study population were 12.1 and 30 months, respectively.

**Table 1:** Patient characteristics at baseline.

Pretreatment								
Characteristics		N = 232 (%)	NLR < 3.19 N = 115 (%)	NLR ≥ 3.19 N = 117 (%)	P (χ^2^)^1^	SII < 810 N = 116 (%)	SII ≥ 810 N = 116 (%)	P (χ^2^)^1^
Sex	Men	142 (61.0)	69 (60.0)	73 (62.4)	0.708	71 (61.2)	71 (61.2)	1.000
	Women	90 (39.0)	46 (40.0)	44 (37.6)		45 (38.8)	45 (38.8)	
Age Distribution	<65	134 (58.0)	62 (53.9)	72 (61.5)	0.240	60 (51.7)	74 (63.8)	0.063
	≥65	98 (42.0)	53 (46.1)	45 (38.5)		56 (48.3)	42 (36.2)	
Primary tumor sidedness	Right	44 (19.0)	21 (18.3)	23 (19.7)	0.786	19 (16.4)	25 (21.6)	0.315
	Left	188 (81.0)	94 (81.7)	94 (80.3)		97 (83.6)	91 (78.4)	
RAS status	Wild Type	95 (40.9)	45 (39.1)	50 (42.7)	0.944	46 (39.7)	49 (42.2)	0.980
	RAS Mutated	99 (42.7)	50 (43.5)	49 (41.9)		51 (44.0)	48 (41.4)	
	Not Tested	32 (13.8)	17 (14.8)	15 (12.8)		16 (13.8)	16 (13.8)	
	BRAF Mutated	6 (2.6)	3 (2.6)	3 (2.6)		3 (2.6)	3 (2.6)	
ECOG PS	0	187 (80.0)	97 (84,3)	90 (76.9)	0.153	95 (81.9)	92 (79.3)	0.618
	≥1	45 (20.0)	18 (15.7)	27 (23.1)		21 (18.1)	24 (20.7)	
Local Ablative Treatment	Yes	81 (35.0)	36 (31.3)	45 (38.5)	0.253	35 (30.2)	46 (39.7)	0.130
	No	151 (65.0)	79 (68.7)	72 (61.5)		81 (69.8)	70 (60.3)	
Targeted Therapy	None	34 (14.6)	19 (16.5)	15 (12.8)	0.284	19 (16.4)	15 (12.9)	0.478
	Bevacizumab	115 (49.6)	51 (44.3)	64 (54.7)		53 (45.7)	62 (53.5)	
	AntiEGFR	83 (35.8)	45 (39.1)	38 (32.5)		44 (37.9)	39 (33.6)	
Chemotherapy	Oxaliplatin	96 (41.3)	47 (40.9)	49 (41.9)	0.365	50 (43.1)	46 (39.7)	0.753
	Irinotecan	133 (57.3)	66 (57.4)	67 (57.3)		65 (56.0)	68 (58.6)	
	Other	3 (1.4)	2 (1.7)	1 (0.8)		1 (0.9)	2 (1.7)	
Primary Tumor Resected	Yes	202 (87.0)	104 (90.4)	98 (83.8)	0.130	106 (91.4)	96 (82.8)	0.050
	No	30 (13.0)	11 (9.6)	19 (16.2)		10 (8.6)	20 (17.2)	
Number of Metastatic Sites	1-2	190 (82.0)	95 (82.6)	95 (81.2)	0.780	97 (83.6)	93 (80.2)	0.495
	>2	42 (18.0)	20 (17.4)	22 (18.8)		19 (16.4)	23 (19.8)	
Liver Metastases	Yes	145 (62.5)	39 (33.9)	106 (90.6)	<0.001	69 (59.5)	76 (65.5)	0.342
	No	87 (37.5)	76 (66.1)	11 (9.4)		47 (40.5)	40 (34.5)	
Peritoneal Metastases	Yes	56 (24.0)	17 (14.8)	39 (33.3)	0.001	26 (22.4)	30 (25.9)	0.539
	No	176 (76.0)	98 (85.2)	78 (66.7)		90 (77.6)	86 (74.1)	

^1^Differences between groups were tested using the χ2 test.

**Table 2:** Best observed response stratified by NLR and SII.

Pretreatment							
Best Response	N = 232 (%)	NLR < 3.19 N = 115 (%)	NLR ≥ 3.19 N = 117 (%)	P (χ^2^)^1^	SII < 810 N = 116 (%)	SII ≥ 810 N = 116 (%)	P (χ^2^)^1^
PR or SD	187 (80.6)	102 (87.9)	85 (72.6)	0.002	100 (86,20)	87 (75)	0.031
PD	45 (16.4)	13 (12.1)	32 (27.4)		16 (13,80)	29 (25)	

PR, partial response; SD, stable disease; PD, progressive disease.

^1^Differences between groups were tested using the χ2 test.

### 3.2 Survival analysis

**Figure 2:**
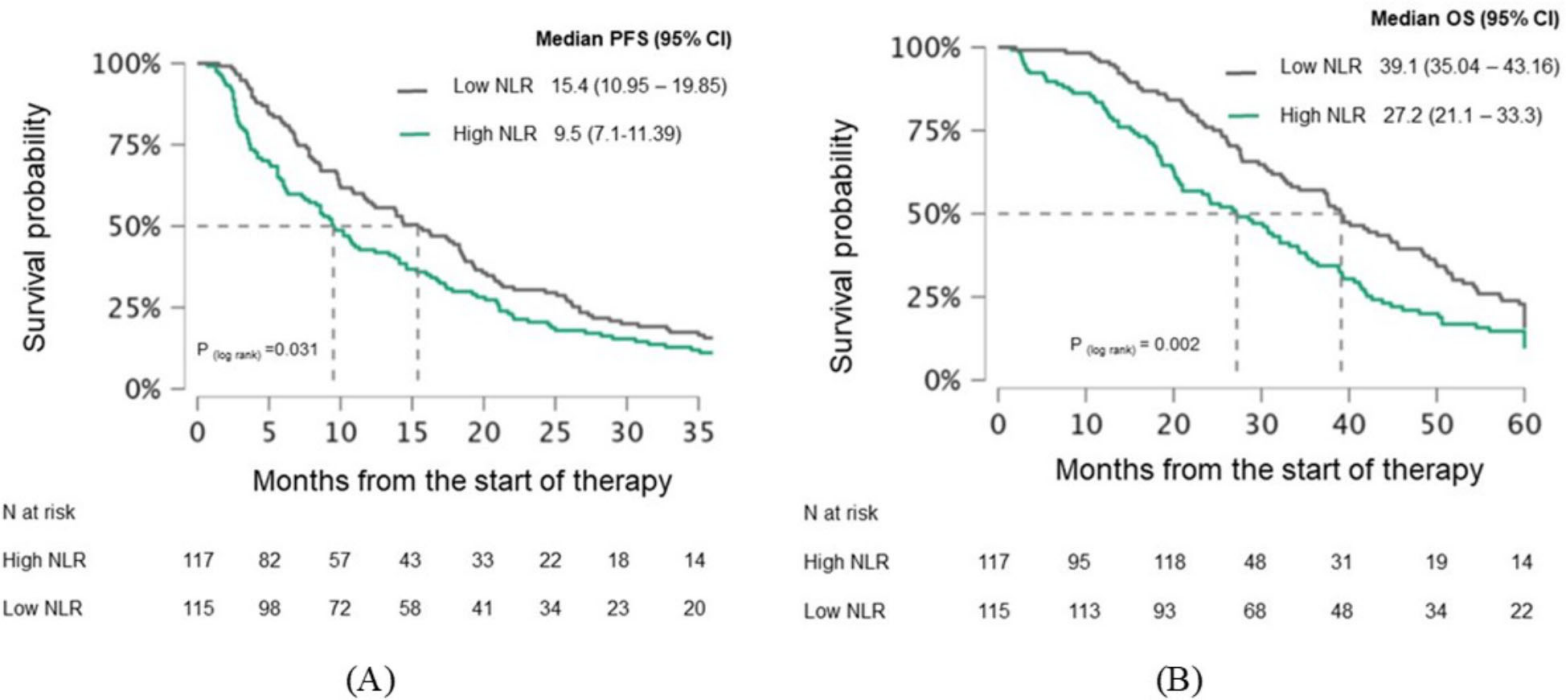
Kaplan-Meier curves demonstrating **(A)** PFS and **(B)** OS in mCRC patients stratified by pretreatment NLR. Curves are shown separately for patients with low NLR and high NLR. Median survival times with 95% confidence intervals **(CI)** and log−rank p−values are indicated on the plots. The numbers of patients at risk for each group are shown in the table below. PFS, Progression-free survival; OS, Overall survival; CI, Confidence interval; NLR, Neutrophil-lymphocyte ratio.

**Figure 3:**
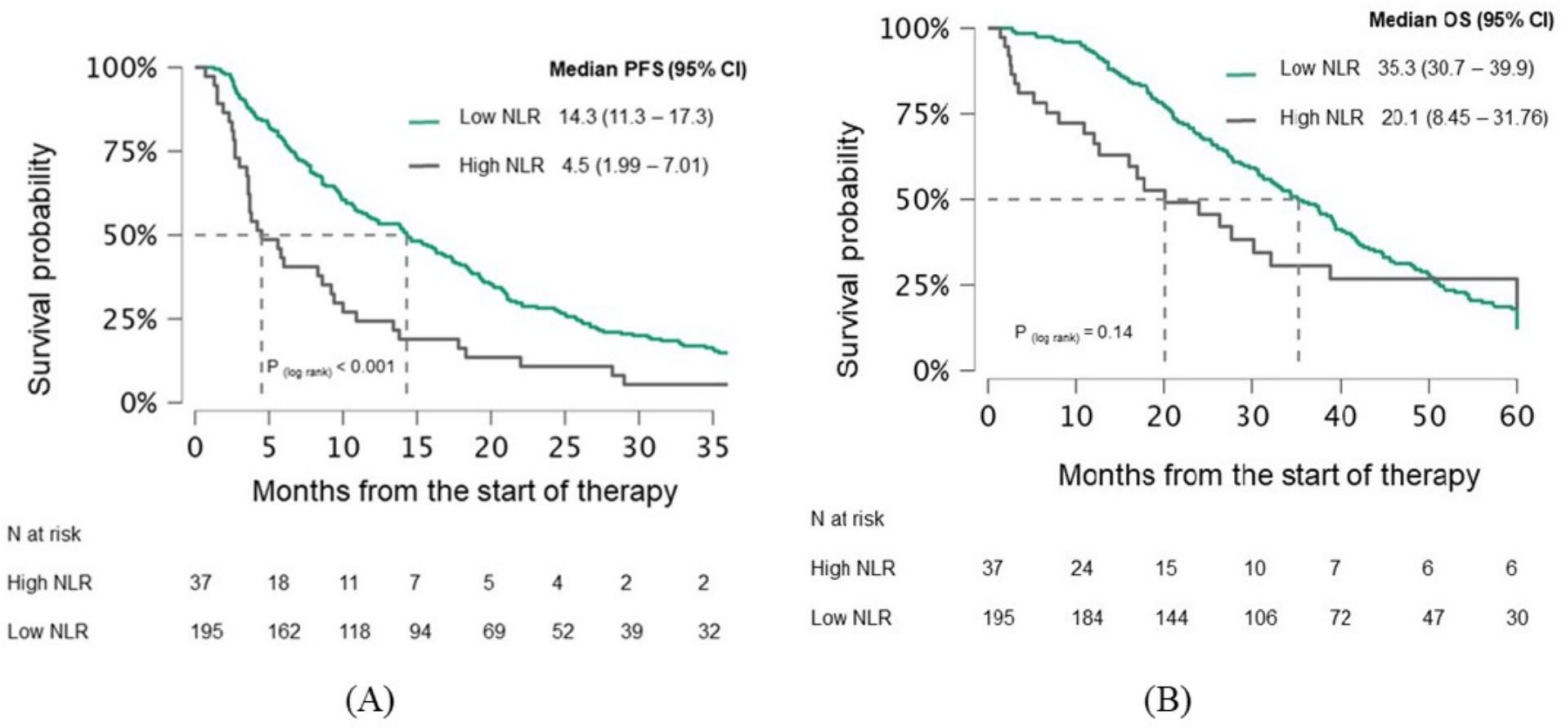
Kaplan-Meier curves demonstrating **(A)** PFS and **(B)** OS in mCRC patients stratified by after 3 months of treatment NLR. Curves are shown separately for patients with low NLR and high NLR. Median survival times with 95% confidence intervals **(CI)** and log−rank p−values are indicated on the plots. The numbers of patients at risk for each group are shown in the table below. PFS, Progression-free survival; OS, Overall survival; CI, Confidence interval; NLR, Neutrophil-lymphocyte ratio.

Patients were divided into two groups based on a pre-treatment median NLR of 3.19: a high NLR group and a low NLR group. PFS was significantly better in the low NLR group compared to the high NLR group (15.4 vs. 9.5 months, p=0.031), as was OS (39.1 vs. 27.2 months, p=0.002) (**Figures 2A, B**). The difference in PFS became even more pronounced after 3 months of treatment, with the low NLR group showing a PFS of 14.3 months compared to 4.5 months in the high NLR group (p<0.001). While the low NLR group also demonstrated longer OS (35.3 vs. 20.1 months), this difference was not statistically significant (p=0.14) (**Figures 3A, B**).

**Figure 4:**
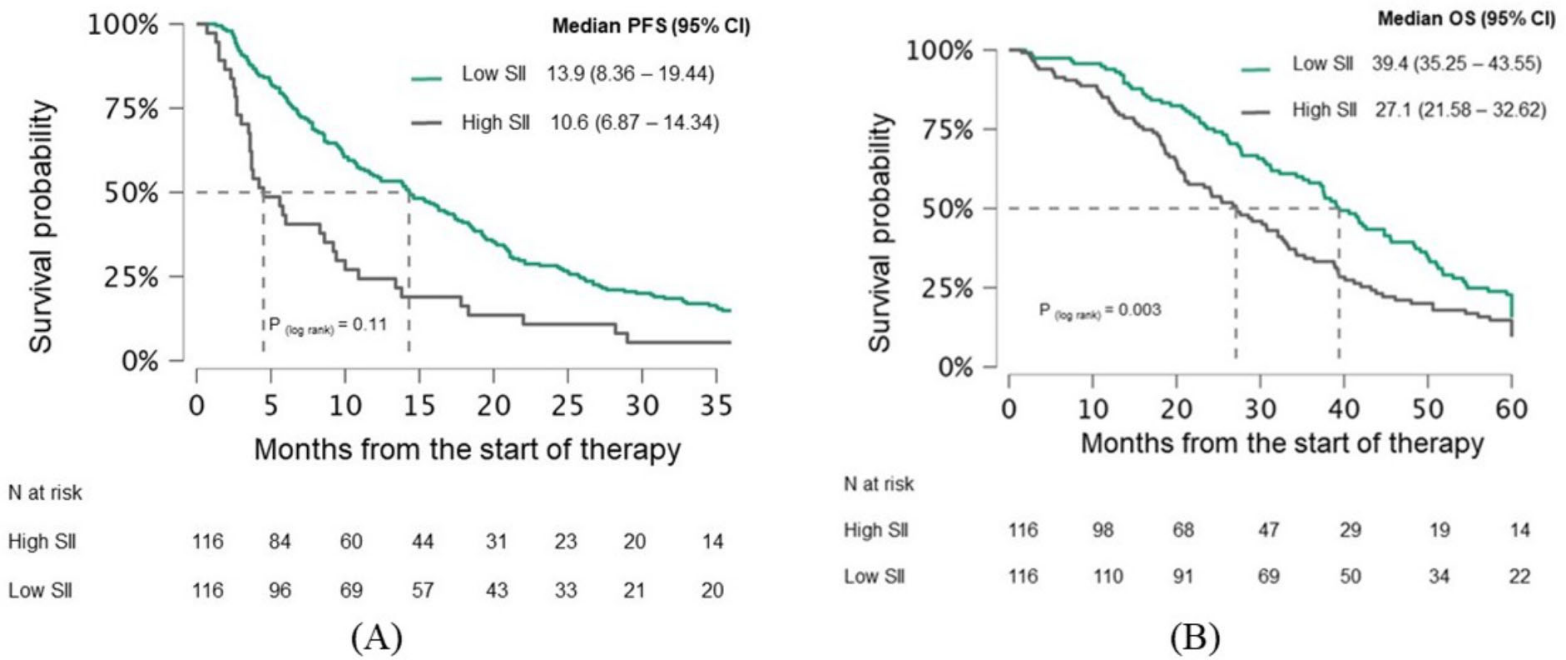
Kaplan-Meier curves demonstrating **(A)** PFS and **(B)** OS in mCRC patients stratified by pretreatment SII. Curves are shown separately for patients with low SII and high SII. Median survival times with 95% confidence intervals **(CI)** and log−rank p−values are indicated on the plots. The numbers of patients at risk for each group are shown in the table below. PFS, Progression-free survival; OS, Overall survival; CI, Confidence interval; SII, systemic immune-inflammation index.

**Figure 5:**
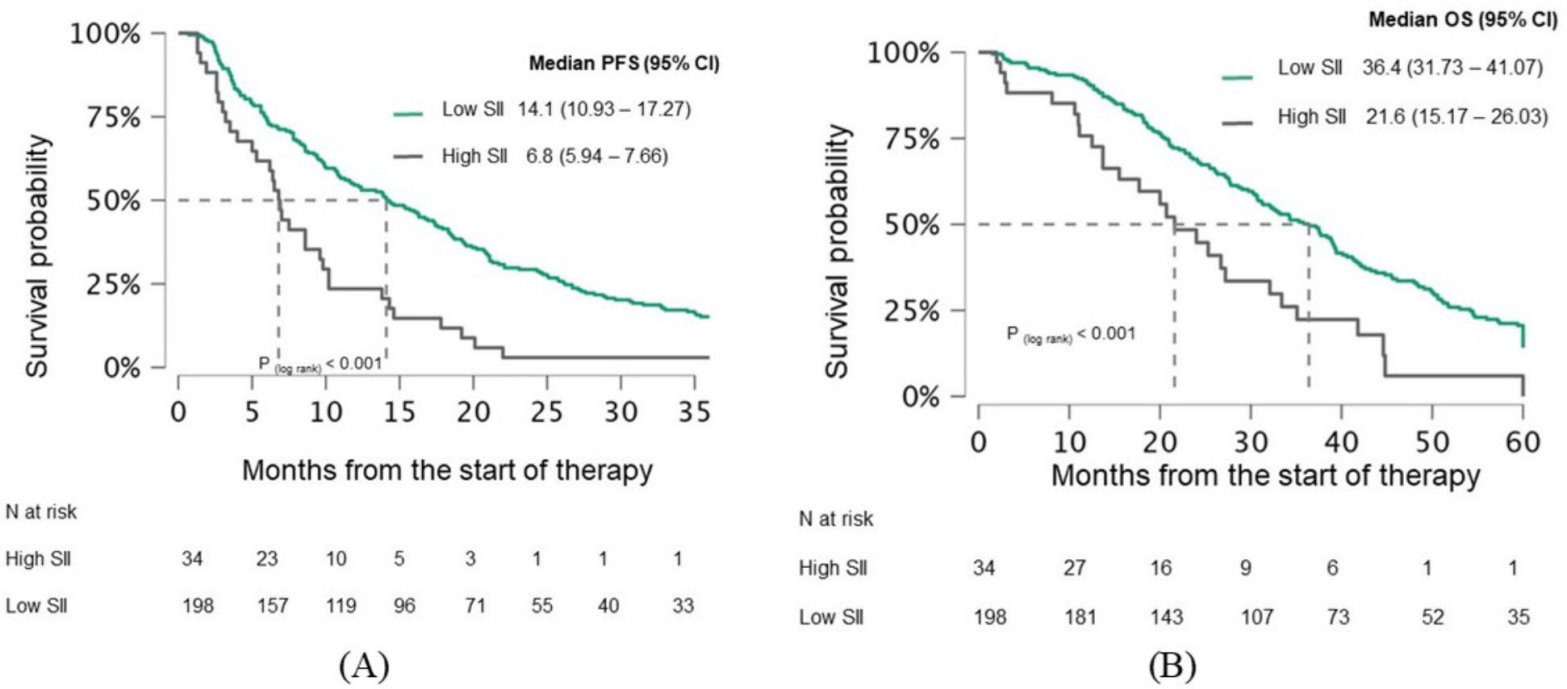
Kaplan-Meier curves demonstrating **(A)** PFS and **(B)** OS in mCRC patients stratified by after 3 months of treatment SII. Curves are shown separately for patients with low SII and high SII. Median survival times with 95% confidence intervals **(CI)** and log−rank p−values are indicated on the plots. The numbers of patients at risk for each group are shown in the table below. PFS, Progression-free survival; OS, Overall survival; CI, Confidence interval; SII, systemic immune-inflammation index.

When patients were categorized based on a median pretreatment SII of 810, there was no statistically significant difference in PFS between the high and low groups (13.9 vs. 10.6 months, p=0.11) (**Figure 4A**). However, a significant difference in OS was observed (39.4 vs. 27.1 months, p=0.003) (**Figure 4B**). After three months of therapy, both PFS (14.1 vs. 6.8 months, p<0.001) and OS (36.4 months vs. 21.6 months, p=0.001) were significantly better in the low SII group (**Figures 5A, B**).

### 3.3 Exploratory analysis of the targeted therapy effect on NLR and SII

To further analyze impact of targeted therapy, patients were divided into three treatment subgroups: chemotherapy without targeted therapy (NE), anti-EGFR (epidermal growth factor receptor) therapy, and anti-VEGF (vascular endothelial growth factor) therapy. Median PFS was 4.7 (95% CI 3.5–6.7) months in the NE group, 16.3 months (95% CI 12.4–21.7) in the anti-EGFR group, and 13.9 months (95% CI 10.2–18.3) in the anti-VEGF group. The global log-rank test showed a statistically significant difference in patients receiving targeted therapy (log-rank p < 0.001). Median OS was 19.1 months (95% CI 15.9–36.4) in the NE group, 39.1 months (95% CI 31.3–45.6) in the anti-EGFR group, and 33.4 months (95% CI 27.7–39.2) in the anti-VEGF group.(log-rank p = 0.018). These findings indicate that PFS and OS do not differ significantly between anti-EGFR and anti-VEGF therapy and that patients who did not receive targeted therapy have poorer outcomes within the limitations of our retrospective cohort. In the exploratory analysis of post-therapy NLR and post-therapy SII, the normality of data distributions was assessed using the Shapiro-Wilks test, which indicated non-normal distributions for both parameters. Consequently, the Mann-Whitney *U* test was employed for group comparisons. The results demonstrated no statistically significant differences in NLR values at the first post-therapy evaluation between patients who received biological therapy and those who did not (P = 0.281), nor between those treated with bevacizumab and those treated with EGFR inhibitors (P = 0.554). There was also no difference when the same analysis was done for SII values (P = 0.688 and P = 0.650, respectively). Overall, these results suggest that neither the administration nor the type of biological therapy was associated with significant changes in post-therapy NLR or SII in this cohort.

### 3.4 Cox proportional hazards analysis

Univariate analyses were performed to identify variables associated with PFS and OS (**Table 3**). In univariate Cox regression analysis, factors associated with PFS and OS were the presence of a BRAF mutation or unknown RAS/BRAF status, application of targeted therapy, number of metastatic sites, receipt of local ablative therapy, and tumor sidedness. Liver metastases were significantly associated with PFS, while resection of the primary tumor and ECOG PS were significantly associated with OS. Only the variables that were significant prognostic parameters in the univariate Cox’s proportional hazards model were included in the multivariate analysis to identify independent prognostic factors for PFS and OS. Cox regression analysis (**Tables 4**–**7**) demonstrated that neither low pretreatment NLR nor SII was associated with improved PFS; however, they were associated with improved OS. Regarding the values after 3 months, low NLR was associated with improved PFS but not OS. After 3 months, low SII was associated with both improved OS and PFS. Patients with ≤2 metastatic sites and those who received local ablative therapy for metastases demonstrated significantly longer PFS and OS. Longer OS was also observed among patients with left-sided primary tumors. In contrast, patients who did not receive targeted therapy in addition to chemotherapy and those with liver metastases showed significantly shorter PFS, though OS was not affected. Resection of the primary tumor does not show a significant impact on OS when analyzed with pre-treatment NLR or SII, but in the analysis after 3 months, NLR or SII has a significant impact on OS. No significant associations were found with either PFS or OS for age, sex, ECOG performance status, RAS mutation status, type of targeted therapy, or peritoneal involvement.

**Table 3:** Univariate Cox proportional hazard models for progression-free survival and overall survival.

Variables		PFS				OS			
		HR	95% CI		P value	HR	95% CI		P value
			Lower	Upper			Lower	Upper	
Sex	Men	1 (ref)				1 (ref)			
	Women	1.043	0,786	1.383	0.771	0.893	0.662	1.206	0.462
Age	≥ 65	1 (ref)				1 (ref)			
	< 65	1.005	0.989	1.021	0.548	1.003	0.986	1.02	0.753
RAS status	wild type	1 (ref)				1 (ref)			
	mutated	1.276	0.937	1.738	0.122	1.177	0.855	1.621	0.318
	BRAF	3.5	1.5	8.16	0.004	5.036	2.146	11.818	< 0.001
	not tested	2.991	1.952	4.582	< 0.001	1.88	1.19	2.97	0.007
Targeted Therapy	anti EGFR	1 (ref)				1 (ref)			
	bevacizumab	1.253	0.919	1.71	0.154	1.186	0.858	1.64	0.301
	none	4.172	2.702	6.441	< 0.001	1.867	1.199	2.908	0.006
Number of Metastatic Sites	1-2	0.509	0.358	0.724	< 0.001	0.516	0.357	0.744	< 0.001
	> 2	1 (ref)				1 (ref)			
Primary tumor sidedness	Left	1 (ref)				1 (ref)			
	Right	1.533	1.085	2.166	0.015	1.687	1.173	2.426	0.005
Primary Tumor Resected	Yes	1 (ref)				1 (ref)			
	No	1.257	0.838	1.886	0.27	1.842	1.213	2.798	0.004
Local Ablative Treatment	Yes	1 (ref)				1 (ref)			
	No	2.031	1.497	2.754	< 0.001	2.054	1.501	2.81	< 0.001
Peritoneal metastases	Yes	1 (ref)				1 (ref)			
	No	0.813	0.590	1.121	0.207	0.871	0.627	1.238	0.465
Liver metastases	Yes	1 (ref)				1 (ref)			
	No	0.738	0.553	0.986	0.04	0.874	0.648	1.178	0.377
ECOG PS	0	1 (ref)				1 (ref)			
	≥ 1	0.764	0.54	1.081	0.128	0.632	0.437	0.914	0.015
NLR pretreatment	≥ 3.19	1 (ref)				1 (ref)			
	< 3.19	0.739	0.56	0.974	0.032	0.639	0.478	0.856	0.003
NLR after 3 months	≥ 3.19	1 (ref)				1 (ref)			
	< 3.19	0.476	0.33	0.687	< 0.001	0.74	0.484	1.12	0.163
SII pretreatment	≥ 810	1 (ref)				1 (ref)			
	< 810	0.799	0.605	1.054	0.112	0.647	0.483	0.866	0.003
SII after 3 months	≥ 810	1 (ref)				1 (ref)			
	< 810	0.44	0.3	0.644	< 0.001	0.485	0.32	0.736	0.001

HR, Hazard ratio; CI, Confidence interval.

**Table 4:** Cox multiple regression model for progression-free survival as outcome for pretreatment and after 3 months of treatment NLR.

Variables		Pretreatment				After 3 months			
		HR	95% CI		P value	HR	95% CI		P value
			Lower	Upper			Lower	Upper	
RAS status	wild type	1 (ref)				1 (ref)			
	mutated	0.896	0.477	1.685	0.734	1.095	0.58	2.068	0.78
	BRAF	1.944	0.728	5.188	0.184	1.250	0.905	1.727	0.176
	not tested	1.457	0.724	2.93	0.291	1.795	0.888	3.63	0.103
Targeted therapy	antiEGFR	1 (ref)				1 (ref)			
	bevacizumab	1.18	0.613	2.272	0.62	0.993	0.511	1.93	0,983
	none	3.877	1.88	7.992	< 0.001	2.979	1.427	6.218	0.004
Primary tumor sidedness	Left	1 (ref)				1 (ref)			
	Right	1.238	0.853	1.795	0.261	1.293	0.891	1.876	0.176
Local Ablative Treatment	Yes	1 (ref)				1 (ref)			
	No	1.939	1.41	2.665	< 0.001	1.987	1.451	2.722	< 0.001
Liver metastases	Yes	1 (ref)				1 (ref)			
	No	0.707	0.514	0.972	0.033	0.693	0.506	0.949	0.022
Number of Metastatic Sites	1-2	0.63	0.423	0.94	0.024	0.666	0.45	1.986	0.042
	> 2	1 (ref)				1 (ref)			
NLR	≥ 3.19	1 (ref)				1 (ref)			
	< 3.19	0.785	0.587	1.05	0.103	0.564	0.383	0.831	0.004

HR, Hazard ratio; CI, Confidence interval.

**Table 5:** Cox multiple regression model for overall survival as outcome for pretreatment and after 3 months of treatment NLR.

Variables		Pretreatment				After 3 months			
		HR	95% CI		P value	HR	95% CI		P value
			Lower	Upper			Lower	Upper	
RAS status	wild type	1 (ref)				1 (ref)			
	mutated	1.161	0-597	2.259	0.66	1.316	0.684	2.53	0.41
	BRAF	2.715	0.937	7.865	0.066	2.738	0.979	7.654	0.055
	not tested	1.345	0.658	2.746	0.416	1.462	0.722	2.96	0.292
Targeted therapy	antiEGFR	1 (ref)				1 (ref)			
	bevacizumab	0.823	0.412	1.646	0.582	0.72	0.367	1.414	0.341
	none	1.4	0.706	2.78	0.336	1.232	0.61	2.488	0.56
Primary tumor sidedness	Left	1 (ref)				1 (ref)			
	Right	1.639	1.08	2.486	0.02	1.663	1.102	2.511	0.015
Primary Tumor Resected	Yes	1 (ref)				1 (ref)			
	No	1.471	0.939	2.307	0.092	1.627	1.042	2.54	0.032
Local Ablative Treatment	Yes	1 (ref)				1 (ref)			
	No	1.929	1.378	2.701	< 0.001	1.931	1.381	2.702	< 0.001
ECOG PS	0	1 (ref)				1 (ref)			
	≥ 1	0.829	0.561	1.225	0.347	0.79	0.536	1.164	0.234
Number of Metastatic Sites	1-2	0.574	0.386	0.854	0.006	0.604	0.407	0.896	0.012
	> 2	1 (ref)				1 (ref)			
NLR	≥ 3.19	1 (ref)				1 (ref)			
	< 3.19	0.65	0.48	0.879	0.005	0.94	0.59	1.496	0.793

HR, Hazard ratio; CI, Confidence interval.

**Table 6:** Cox multiple regression model for progression-free survival as outcome for pretreatment and after 3 months of treatment SII.

Variables		Pretreatment				After 3 months			
		HR	95% CI		P value	HR	95% CI		P value
			Lower	Upper			Lower	Upper	
RAS status	wild type	1 (ref)				1 (ref)			
	mutated	0.869	0.949	0.509	0.869	0.899	0.489	1.653	0.732
	BRAF	2.056	0.776	5.445	0.147	2.056	1.841	0.716	0.205
	not tested	1.463	0.729	2,935	0.285	1.348	0.678	2.68	0.395
Targeted therapy	antiEGFR	1 (ref)				1 (ref)			
	bevacizumab	1.152	0.601	2.208	0.669	1.147	0.61	2.158	0.67
	none	3.875	1.885	7.967	< 0.001	3.79	1.881	7.635	< 0.001
Primary tumor sidedness	Left	1 (ref)				1 (ref)			
	Right	1.207	0.833	1.751	0.32	1.214	0.84	1.753	0.301
Local Ablative Treatment	Yes	1 (ref)				1 (ref)			
	No	1.969	1.434	2.704	< 0.001	1.965	1.428	2.702	< 0.001
Liver metastases	Yes	1 (ref)				1 (ref)			
	No	0.684	0.499	0.936	0.018	0.713	0.518	0.981	0.038
Number of Metastatic Sites	1-2	0.643	0.432	0.957	0.03	0.673	0.454	0.998	0.049
	> 2	1 (ref)				1 (ref)			
SII	≥ 810	1 (ref)				1 (ref)			
	< 810	0.823	0.619	1.094	0.18	0.653	0.436	0.977	0.03

HR, Hazard ratio; CI, Confidence interval.

**Table 7:** Cox multiple regression model for overall survival as outcome for pretreatment and after 3 months of treatment SII.

Variables		Pretreatment				After 3 months			
		HR	95% CI		P value	HR	95% CI		P value
			Lower	Upper			Lower	Upper	
RAS status	wild type	1 (ref)				1 (ref)			
	mutated	1.296	0.673	2.496	0.438	1.235	0.653	2.333	0.516
	BRAF	3.066	1.071	8.778	0.037	2.541	0.924	6.989	0.071
	not tested	1.459	0.718	2.969	0.297	1.397	0.701	2.786	0.342
Targeted therapy	antiEGFR	1 (ref)				1 (ref)			
	bevacizumab	0.757	0.383	1.496	0.423	0.743	0.385	1.435	0.376
	none	1.286	0.654	2.529	0.466	1.232	0.636	2.386	0.535
Primary tumor sidedness	Left	1 (ref)				1 (ref)			
	Right	1.587	1.052	2.396	0.028	1.631	1.081	2.461	0.02
Primary Tumor Resected	Yes	1 (ref)				1 (ref)			
	No	1.394	0.883	2.199	0.154	1.687	1.077	2.643	0.022
Local Ablative Treatment	Yes	1 (ref)				1 (ref)			
	No	2.021	1.446	2.824	< 0.001	1.929	1.378	2.699	< 0.001
ECOG PS	0	1 (ref)				1 (ref)			
	≥ 1	0.783	0.533	1.151	0.213	0.878	0.586	1.316	0.528
Number of Metastatic Sites	1-2	0.557	0.374	0.83	0.004	0.619	0.417	0.92	0.018
	> 2	1 (ref)				1 (ref)			
SII	≥ 810	1 (ref)				1 (ref)			
	< 810	0.596	0.439	0.81	0.001	0.632	0.404	0.987	0.044

HR, Hazard ratio; CI, Confidence interval.

## 4 Discussion

Cancer-associated inflammation plays a significant role in disease progression and is linked to poor outcomes in patients undergoing systemic therapy for metastatic disease (24). This process involves various inflammatory cells that produce inflammatory mediators. Neutrophils can support tumor growth, angiogenesis, and metastasis, and may also contribute to treatment resistance. They are functionally plastic and can adopt either pro-tumor (N2) or anti-tumor (N1) phenotypes, depending on the tumor microenvironment. Platelets similarly promote angiogenesis and help tumor cells evade immune surveillance. In contrast, lymphocytes are crucial for anti-tumor immunity and the clearance of tumor cells (25). While many inflammatory markers have been studied for their prognostic significance, there is still insufficient data on their predictive value in patients receiving first-line treatment for mCRC. NLR was the first immune-related marker extensively researched in various tumors, showing in CRC to be predictive of survival (26). Also, it was reported as an independent prognostic factor in patients receiving first-line chemotherapy for mCRC (27). SII is a promising biomarker that incorporates neutrophils, platelets, and lymphocytes into a single score, where high values may indicate a pro-tumor inflammatory state with a compromised immune response (16).

In this study, we conducted serial assessments of NLR and SII to investigate their prognostic significance for survival outcomes and treatment response among 232 patients with mCRC who received first-line chemotherapy, with targeted therapy added in most cases (85.4%). Specifically, we analyzed both baseline (pretreatment) and 3-month post-treatment values of NLR and SII to determine their prognostic value. Our results indicate that post-treatment NLR and especially SII are stronger prognostic factors of survival, particularly PFS on first-line chemotherapy. Patients with high NLR and SII after treatment had significantly shorter PFS compared to those with lower values. Multivariate analysis confirmed that pretreatment NLR and SII are independent prognostic factors for OS, but not for PFS. Regarding post-treatment assessment, NLR appears to be an independent prognostic factor for PFS, but not for OS. Post-treatment SII is independently associated with improved OS and PFS. This suggests that post-treatment values of NLR and even more of SII better reflect treatment response and survival outcomes than baseline values alone. Therefore, serial assessment of NLR and SII early during treatment could provide valuable prognostic information and support more timely and informed therapeutic decisions.

Similar findings have been reported by Liu et al. with NLR on a smaller cohort of patients (n = 127) receiving FOLFOX chemotherapy for mCRC, showing that elevated baseline NLR was associated with poor PFS, but not OS. However, the change from high to low post-treatment NLR values was associated with treatment benefit and improved survival. Patients included in this study did not receive any targeted therapy (28). In a study conducted by Kim et al., the pattern of change in NLR was evaluated, demonstrating that a reduction in NLR served as an independent predictor of chemotherapy response and improved survival outcomes. Conversely, a persistently elevated NLR, or a shift from a low to a high NLR, was associated with poorer survival. This study focused on the chemotherapy regimen and did not explore the prognostic role of targeted therapy (19). Similarly, another study involving 71 patients investigated the dynamic changes in inflammatory indices (NLR, platelet-to-lymphocyte ratio, PLR, lymphocyte-to-monocyte ratio, LMR) during first-line chemotherapy and their prognostic significance in patients with mCRC. This study found that among the evaluated indices, only NLR was significantly correlated with PFS and OS, while no association was observed between NLR dynamics and response to treatment. The prognostic value of SII has not been investigated in this research (29). In study by Kocak et al., prognostic value for dynamic changes in NLR or PLR was not observed, while improvements in the prognostic nutritional index (PNI) were associated with longer survival. The PNI, calculated from serum albumin and lymphocyte count, reflects the patient’s immunonutritional status and has repeatedly been shown to correlate with outcomes in mCRC, with higher values indicating better treatment tolerance and more favorable tumor biology (30). Although our study did not specifically analyze dynamic changes, our findings align with these observations by demonstrating that post-treatment inflammatory status has greater prognostic significance than pretreatment levels alone, underscoring the need for longitudinal assessment.

While our results were independent of RAS/BRAF mutational status, conflicting data from Miyamoto et al. suggest that systemic inflammation biomarkers are predictive in the KRAS wild-type population but not in the KRAS-mutated population. However, this study was unable to conclude whether the selection of targeted therapy was effective (31). On the other hand, two retrospective studies concluded that pre-treatment inflammatory indices can predict the efficacy of cetuximab therapy, with elevated NLR being associated with shorter PFS and OS. Other indices, including the SII, did not demonstrate a statistically significant correlation with survival outcomes. Both studies lack data on post-treatment values and focus on a highly selected mCRC population – RAS wild-type patients treated with cetuximab (32, 33). Conversely, Passardi et al. identified both SII and, more prominently, NLR as prognostic and predictive biomarkers of survival in patients treated with chemotherapy plus bevacizumab in two separate studies. Notably, none of the aforementioned studies assessed the prognostic value of post-treatment inflammatory indices in relation to survival (34, 35). Anti-VEGF therapy can reduce tumor-driven angiogenesis and indirectly attenuate pro-inflammatory signaling, while anti-EGFR therapy may alter tumor cell proliferation and downstream cytokine production (36). However, despite these potential differences, no significant variation in PFS or OS was observed between patients receiving anti-VEGF therapy or anti-EGFR therapy in our cohort. Likewise, the distribution of NLR and SII at baseline and during follow-up did not differ meaningfully between these groups. In our study, SII appeared to be a more robust prognostic factor than NLR. An improvement in PFS was observed in patients receiving targeted therapy, with no benefit in OS. However, this could be influenced by other factors not evaluated in this study, as patients who did not receive targeted therapy may have had contraindications for it. Low pre-treatment NLR and SII were associated with a better response to treatment.

A recent meta-analysis has demonstrated that SII is linked to poorer outcomes in patients with CRC. However, in stage IV patients, the association was not statistically significant. Nonetheless, none of the included studies assessed the temporal patterns of inflammatory biomarkers or the potential impact of treatment on outcomes. The authors suggest that longitudinal assessment in future research could improve the utility of inflammatory indices (20). Conversely, Tan et al. conducted a meta−analysis showing that pre−treatment SII was more strongly associated with both PFS and OS than post−treatment values, suggesting that the prognostic impact of SII may be attenuated following therapy. The studies included in the analysis were heterogeneous, spanning stages I-IV, and only 6 out of the 27 studies investigated chemotherapy combined with targeted therapy as the treatment modality (37). In contrast, our findings highlight the importance of serial assessment of NLR and SII in mCRC, supporting the need for stage-specific studies to better define the temporal evolution of these inflammatory markers during systemic treatment.

We also examined other factors that may have an impact on survival and the prognosis of mCRC patients. In our cohort, improvement in OS and PFS was also influenced by the number of metastatic sites and the use of local ablative treatment. Local ablative treatments, including metastasectomy, MWA, and SBRT, may improve outcomes in selected patients with mCRC by reducing overall tumor burden, delaying systemic progression, and extending the interval to subsequent systemic therapy. These interventions are generally reserved for patients with oligometastatic disease, favorable performance status, and favorable tumor biology, which are associated with improved survival. Beyond their cytoreductive effects, local therapies may influence systemic inflammation and alter NLR and SII (38, 39). While this study was not designed to determine the independent effects of local therapy, the potential interaction between local interventions and systemic inflammatory markers should be considered when interpreting the prognostic value of longitudinal NLR and SII trends. Conversely, liver and/or peritoneal involvement did not show an association with OS, although patients with liver involvement had shorter PFS. Liver metastases represent the most prevalent site in mCRC, followed by metastases to the lungs and the peritoneum (40). They are known to negatively impact survival, with the number of lesions affecting the outcome (41). Peritoneal metastases represent adverse prognostic and predictive factors, as patients with this pattern of spread derive limited benefit from systemic chemotherapy (42). Also, patients with left-sided tumors experienced prolonged OS. Resection of the primary tumor had a significant impact on OS only when analyzed with post-treatment NLR and SII, but not in pretreatment assessment. The impact of primary tumor resection on survival in patients with mCRC remains controversial, likely due to the heterogeneity of the populations, although patients with left-sided tumors do experience prolonged survival (43). Moreover, the systemic inflammatory response induced by primary tumor resection may enhance immunosuppression and potentially accelerate metastatic progression (44). Most patients in our study were in good general condition, with an ECOG PS of 0. A poor ECOG PS has been identified as a risk factor for worse survival and increased toxicity, and it is included in treatment guidelines for mCRC (4). However, most studies group ECOG PS 0 and 1 patients as having a good general condition, although patients with ECOG PS 0 show significantly better treatment outcomes, both in terms of survival and a reduction in serious adverse events (45). In our analysis, ECOG PS was associated with improved OS in the univariate analysis, although this association did not remain significant in the multivariate model.

Finally, there is considerable variability in the cutoff values for the NLR across studies, with thresholds ranging from 2 to 5. These thresholds have been established using various methods, such as receiver operating characteristic (ROC) curves, median or mean values from study populations, or reference to previously published data (46). In contrast, cutoff values for the SII are even more limited and heterogeneous, ranging from 340 to over 6000 (17, 37). Although ROC curves provide an objective method for defining cut-offs, their implementation in routine clinical practice remains challenging. Despite consideration of ROC-based cut-offs, the cohort heterogeneity, variability in PFS/OS endpoints, and the absence of a universally validated reference cut-off for NLR/SII in metastatic disease led to limited clinical interpretability. Because our objective was to evaluate prognostic trends rather than define clinically adoptable thresholds, we selected the median as a robust, distribution-based, and non-model-dependent method, which is frequently applied in similar retrospective biomarker studies. Additionally, NLR and SII values may be influenced by factors such as age, race, and comorbidities, further complicating the determination of standardized cutoff values (24). Despite these limitations, NLR and SII possess a prognostic role, and combining them with other validated biomarkers (RAS and BRAF molecular testing, and carcinoembryonic antigen (CEA) monitoring) and clinical parameters (such as ECOG PS, radiological assessment) in routine clinical practice for individual prognosis and treatment selection could enhance long-term prognostic assessments (19, 26). Furthermore, integrating inflammatory biomarkers derived from CBC with multi-omics approaches, such as interleukin-27 receptor alpha (IL27RA) expression, may enhance the assessment of the inflamed tumor microenvironment and its prognostic relevance (47).

Unlike the mentioned studies, which focused predominantly on pre-treatment inflammatory indices, this study emphasizes the prognostic significance of serially assessing NLR and SII at 3 months post-treatment to better reflect treatment response and survival outcomes. Baseline inflammatory markers reflect a composite of tumor-related inflammation, comorbid conditions, and pre-treatment physiological state, and therefore may not accurately represent the biology driving subsequent disease behavior (25). In contrast, post-treatment NLR and SII capture the host response after the first cycles of systemic therapy, integrating both tumor sensitivity to treatment and the degree of treatment-induced or residual systemic inflammation. Patients in whom inflammatory markers decrease after treatment likely experience effective cytoreduction and reduced tumor-driven myelopoiesis, whereas persistently elevated values may indicate ongoing tumor activity, inadequate response, or a sustained pro-tumor inflammatory milieu (19). This dynamic information appears to provide more accurate prognostic insight than baseline measurements alone, which is consistent with the superior predictive performance of 3-month post-treatment NLR and SII in our cohort. By conducting serial assessments of NLR and SII early in the treatment process, we can gain valuable prognostic information that may lead to more timely and informed therapeutic decisions. This aspect has not been extensively explored in previous research.

This study has several limitations that should be acknowledged. The retrospective, single-center design introduces potential selection bias and limits external validity, as patient management and data collection may not reflect broader practice patterns. Heterogeneity in patient characteristics and therapeutic regimens may also confound outcomes. Furthermore, the absence of standardized cut-off values for these markers reduces comparability across studies and may impair generalizability.

## 5 Conclusions

A better understanding of the role of systemic inflammation in the prognosis of mCRC and response to treatment could improve patient selection for a more personalized therapeutic approach. Inflammatory biomarkers derived from CBC are readily available, robust, and cost-effective prognostic factors for outcomes in patients with mCRC. Consequently, there is a need for further investigation into the role of NLR and SII in identifying high-risk patient groups and guiding the treatment of mCRC. To validate the prognostic value of NLR and SII and their dynamics, future prospective trials should focus on specific mCRC patient subpopulations based on disease biology and treatment modalities. Additionally, it should be investigated whether a serial assessment of NLR and SII has prognostic value in later lines of treatment.

## Data Availability

The raw data supporting the conclusions of this article will be made available by the authors, without undue reservation.
